# Theoretical dietary modelling of Australian seafood species to meet long-chain omega 3 fatty acid dietary recommendations

**DOI:** 10.3402/fnr.v57i0.20737

**Published:** 2013-10-29

**Authors:** Jessica A. Grieger, Catherine McLeod, Lily Chan, Michelle D. Miller

**Affiliations:** 1 Nutrition and Dietetics, Flinders University, Adelaide, South Australia; 2 South Australian Research and Development Institute, Adelaide, South Australia

**Keywords:** seafood modelling, older adults, long-chain omega 3 fatty acids, seafood recommendations, Australia

## Abstract

**Background:**

Several agencies recommend seafood to be consumed 2–3 times per week. In Australia, there is a lack of nutrient composition data for seafood species and it is not known whether including different seafood species in a diet would provide sufficient long-chain omega 3 fatty acids (LC *n*–3 PUFA) to meet various national recommendations.

**Objective:**

To utilise recent nutrient composition data for major Australian seafood groups (*n*=24) with the addition of two tuna options (total *n*=26) to: (1) determine whether including these species into a diet based on the Australian Guide to Healthy Eating (AGHE) will achieve LC *n*–3 PUFA recommendations [Adequate Intake (AI: 160 mg/d men, 90 mg/d women)], Suggested Dietary Target (SDT), 500 mg/d Heart Foundation (HF) recommendation and (2) determine the weekly number of servings of seafood to meet recommendations using either lower fat (*n*=23, <10% total fat) or higher fat (*n*=3, ≥10% total fat) seafood.

**Design:**

Two simulation models incorporated all 26 species of seafood or only lower fat seafood into a diet based on the AGHE. Two further models identified the number of servings of lower or higher fat seafood required to meet recommendations.

**Results:**

Including 2 and 3 servings/week of any seafood would enable 89% of women and 66% of men to meet the AI. Including only lower fat seafood would enable 83% of women and 47% of men to meet the AI. Half a serving/week of higher fat seafood would enable 100% of men and women to meet the AI.

**Conclusions:**

Including the recommended 2–3 servings of seafood/week requires at least some higher fat seafood to be consumed in order for most men and women to meet the AI. Further messages and nutrition resources are needed which provide options on how to increase intake of LC *n*–3 PUFA, specifically through consumption of the higher fat seafood.

There is widespread literature reporting the health benefits of increased fish and omega 3 consumption on reducing triacylglycerol concentrations (1), and reducing the risk of all-cause mortality (2) and death from cardiac events (3). In particular, as part of a healthy diet, increased fish consumption has been advised to reduce risk for cardiovascular disease (CVD) (4, 5). Risk of CVD increases with age, and although there has been a steady decline in its prevalence over the last 30 years, it is still one of the biggest causes of mortality and it contributes a large burden of illness and disability globally (6).

For intake of long-chain omega 3 fatty acids (LC *n*–3 PUFA), the National Health and Medical Research Council (NHMRC) in Australia recommends 160 mg/d for men and 90 mg/d for women as the Adequate Intake (AI) (7). This is based on observed or experimentally-determined approximations or estimates of nutrient intake for apparently healthy people (7). The NHMRC also recommends a Suggested Dietary Target (SDT) of 610 mg/d and 430 mg/d for adult men and women, respectively; this level of intake may help in prevention of chronic disease (7). Moreover, the Heart Foundation (HF) of Australia also recommends an intake of 500 mg/d of combined eicosapentanoic acid (EPA) and docosahexanoic acid (DHA) to lower risk of coronary heart disease (4). Thus, there is a range of recommended intakes depending on the health of the individual. These intakes are within the range of values recommended in the Netherlands (8), USA and Canada (9), and France (10).

Regarding the consumption of fish, numerous agencies recommend 2–3 servings of oily fish per week to reduce risk for heart disease (4, 5, 9) and the World Health Organization recommends regular fish consumption of 1–2 servings/week; each serving should provide the equivalent of 200–500 mg EPA+DHA (11). Compared to recommendations, dietary intakes of seafood and LC *n*–3 PUFA in older Australians as well as older adults overseas (12–15) are inadequate. Only two large dietary surveys conducted in Australia have assessed seafood and/or LC *n*–3 PUFA consumption (16, 17). Among the 45–64 year olds participating in the 1995 National Nutrition Survey (NNS) (*n*=4,329), mean consumption of fish and seafood products was estimated to be 32.8 g/d and in those ≥65 years was 25.6 g/d; consumption of finfish (excluding canned fish) was just over 8 g/d for both age groups (17). In the 2007 Australian National Children's Nutrition and Physical Activity Survey (*n*=4,487), mean consumption of fish and seafood products was 10–13 g/d in boys and 11–15 g/d in girls (16). In comparison, a smaller, but recent national survey of 854 Australian adults ≥51 years, identified that 34% ate finfish and/or seafood ≥2 times/week, with a mean intake of total finfish/seafood of 43 g/d and a mean intake of LC *n*–3 PUFA of 508 mg/d (18). In this group, the most frequently consumed types of finfish (≥2 times/week) were salmon and tuna; however, only 28% of respondents (*n*=242) consumed ≥500 mg/d LC *n*–3 PUFA from finfish and/or seafood alone (18). In that same survey, it was found that only 12% of the older adults consumed higher fat fish ≥2 times/week (e.g. salmon, sardines, herring), whereas 22% consumed the lower fat fish types ≥2 times/week (e.g. canned tuna, shark and prawns) (18). Moreover, 60% of the participants identified sardines as being high in oils/fatty acids but only 18% indicated that salmon and trout were high in oils/fatty acids (19). The perception of what types of seafood contain high amounts of omega 3 likely coincides with the greater percentage of adults consuming the lower fat seafood and the small percentage of adults meeting the recommendations for seafood consumption. Enabling consumers to have an understanding of the potential for specific seafood species to assist in meeting LC *n*–3 PUFA recommendations would be valuable in order to assist in increasing the number of adults achieving health benefits through the consumption of seafood.

In Australia, there is a lack of nutrient composition data for various seafood species (20, 21), which makes it difficult to determine the exact amount of LC *n*–3 PUFA consumed. Based on recently completed nutrient profiling of 24 Australian seafood groups (22), it was considered important to identify whether consumption of these different seafood types would provide sufficient LC *n*–3 PUFA to meet current dietary recommendations. Therefore, the objectives of this study were: (1) to utilise recent nutrient composition data for Australian seafood to determine whether including these types of seafood into a diet based on the Australian Guide to Healthy Eating (AGHE) will achieve LC *n*–3 PUFA recommendations (i.e. AI, SDT, 500 mg/d HF recommendation) and (2) to determine the weekly number of lower fat and higher fat servings of seafood required to meet LC *n*–3 PUFA recommendations. Dietary modelling provides information on how combinations of various foods with nutrient composition data can meet nutrient recommendations. Results from the current dietary modelling can inform health professionals on appropriate recommendations for fish consumption (species and frequency of consumption) and how these targets can be achieved.

## Methods

### Overview of approach

Four theoretical dietary modelling procedures were undertaken utilising the nutrient profiling data. Two models involved the incorporation of seafood into a 14-day meal plan consistent with the number of food group servings in the AGHE available for 60+ year olds (Table 1) (23). The older age group was chosen due to the frequent low intake of seafood (12–15, 18) and their higher prevalence of heart disease (6). Theoretical energy and macronutrient intakes from these diets were compared against current Nutrient Reference Values (NRVs) for 51–70 year olds (7). Two further models were carried out to identify the number of weekly servings of lower fat and higher fat seafood required to meet LC *n*–3 PUFA recommendations.

**Table 1:** Number of food group servings from the Australian Guide to Healthy Eating for those aged 60 years and over

		Breads and cereals^a^	Vegetable^b^	Fruit^c^	Dairy^d^	Meat and alternatives^e^	Extras^f^
Men	AGHE	4–6	4–7	2–3	2–3	1–1.5	0–2.5
	Dietary modelling	5.9	6.1	2.4	2.5	1.5	2.5
Women	AGHE	3–5	4–6	2–3	2–3	1–1.5	0–2
	Dietary modelling	4.5	5.0	2.0	3.1	1.5	2.0

a Serving size equivalents: ^a^30 g crackers; 1 slice bread (40 g); 30 g cereal; 90 g dry weight rice/pasta

b 75 g all vegetables

c 150 g medium-piece fresh fruit; 125ml juice; 150 g diced/canned fruit

d 250 ml milk; 200 g yoghurt; 40 g cheese

e 100 g cooked meat, poultry, fish; 75 g cooked peas; beans, lentils, 1/3 cup nuts; 1/4 cup seeds

f 20 g oil; 1 serve ∼600 kJ.

### AGHE and nutrient recommendations

The AGHE was used to build the 14-day menu based on food group guidelines for 60+ year olds. The AGHE was developed in 1998 and the meal plan was devised so that the different gender and age groups could meet 70% of the Recommended Daily Intake (RDI) (i.e. RDIs established in 1990). As new NRVs have since been made (7), the current study used the AGHE as the basis for a 14-day meal plan; however, assessment of the theoretical diet was assessed against current NRVs for men and women aged 51–70 years. Specifically, macronutrient recommendations were based on the Acceptable Macronutrient Distribution (AMDR) for protein, carbohydrates, and fat and/or AI for fibre and LC *n*–3 PUFA (160 mg/d men, 90 mg/d women) (7). The SDT (SDT: 430 mg/d women, 610 mg/d men) (7) and the HF recommendation of 500 mg/d EPA+DHA (4) were also used in determining the percentage of adults who would meet these LC *n*–3 PUFA targets.

### Food groups and foods

Commonly consumed foods (*n*=207) were chosen from the AUSNUT database and their energy and nutrient content was extracted using Food Works (FoodWorks 2009, version 6.0.2539, Xyris Software Brisbane). Twenty-six foods were included in the breads and cereals group, in which 11 were white/plain/refined options, 12 were wholegrain/wholemeal options and 3 were classed as ‘other’ (e.g. fruit bread, crackers). The number of individual foods in the breads and cereals food group was based on the percentage that these foods were consumed by adults >19 years from the 1995 NNS. For example, ‘bread and bread rolls’ provided approximately 40% of mean daily intake for breads and cereals in that survey (17). Therefore, 8 choices of bread and bread rolls were included in the breads and cereals category. For the 40 vegetable options, 11 were starchy vegetables (potato and sweet corn), 7 were orange/red vegetables (carrot, pumpkin and sweet potato), 5 were darker green and brassica vegetables (broccoli, spinach, green bean, cauliflower and cabbage), and 17 were other types of vegetables (celery, cucumber, lettuce, mushroom, onion, beetroot, capsicum, squash, tomato, broad bean, snow pea, parsnip and zucchini). For the 22 fruit options, 16 were fresh (4 were apples and pears), 3 options were fruit juices, and 3 were canned varieties. Within the 26 dairy options, 11 were various milk types (e.g. skim, low fat, full fat), 8 were cheese, 4 were yoghurt and 3 were other (custard, ice cream, condensed milk). For the meat and alternative options, 14 were red meat (including veal and pork), 6 white meat (chicken and turkey), 7 beans/legumes, 3 options for eggs, and 7 options for nuts and seeds. Sausages, bacon, salami-type deli meats were included in the extras category due to their higher energy, fat, and sodium content, even though they were reported in the NNS under the meat and meat dishes group. An option of ice cream was included in the dairy and extras category as it contains calcium, but also can be high in fat/sugar. There were 58 options for the extras category in which the majority contained approximately 600 kJ/serving (e.g. 400 ml regular beer, 596 kJ; 40 g iced apple cake, 607 kJ; 40 g pure cream, 664 kJ; 60 g party sausage roll, 799 kJ), as well as 6 options of unsaturated fats and oil foods (3 margarine, three oils).

### Nutrient profiles of seafood

Twenty-one different seafood species sampled between July 2010 and July 2011, from the 2012 Australian Seafood Compositional Profiles study (22) were included in the models (including both wild and farmed seafood for 3 of the species, totalling 24 different groups of seafood). In the profiles study, most seafood products were analysed raw (except burrowing black fish and the farmed prawn species) and all values were reported in 100 g portions (Table 2). Due to changes in food weight upon cooking, a weight change factor for the raw products was used (24). Weight change factors (reported as a percentage) were listed for ‘finfish, fresh, frozen’ that had either been baked (−24% weight change), grilled (−26%), microwaved (−19%), poached (−16%) and steamed (−17%). A weight change factor of −25% (i.e. 100 g fish–25% loss=75 for calculation) was used as an intermediate between baked and grilled weight loss as this was the most frequently reported method of cooking in a previous study that we conducted (18). In addition, to account for any changes in fatty acid content following weight loss during cooking, a retention factor was used to convert fatty acid content of raw fish to fatty acid content in cooked fish (0.8 for fish with >5% fat content, 0.95 for fish with <5% fat content and 1.0 for crustaceans and molluscs). It was assumed that the change in any particular fatty acid content would be similar to the changes in total fat content upon cooking. As there are no current data on retention factors for fatty acids, values were adopted from retention factors reported in a German publication (25). The content of LC *n*–3 PUFA ranged from 11.3 mg/100 g (cooked burrowing blackfish) to 3,755 mg/100 g (cooked ocean trout). Three of the species were considered to be high fat (‘higher fat’, ≥10% total fat: Atlantic salmon, ocean trout, yellowtail kingfish), according to the HF in Australia (26), one was moderate in total fat (barramundi, 7.4%) and the remaining were considered to be low fat (<5% total fat). The moderate-fat and low-fat seafood species were grouped (‘lower fat’).

**Table 2:** Seafood species, long-chain omega 3 content (per 100 g) and frequencies used in the dietary modelling

Seafood species	Aquaculture/wild	EPA (mg)	DHA (mg)	DPA (mg)	Frequencies of selection (Model 1)	Frequencies of selection (Model 2)
Atlantic Salmon	Aquaculture	1,030	790	372	0.142	–
Australian Sardine	Wild	450	178	21.8	0.014	0.022
Banana Prawn*	Aquaculture	130	83.8	6.25	0.014	0.022
Banana Prawn	Wild	63.4	76.7	10.7	0.014	0.022
Barramundi	Aquaculture	370	401	133	0.057	0.088
Black Lip Abalone	Wild	1.25	37.2	52.2	0.003	0.004
Black Lip Abalone	Aquaculture	18	65.2	68.5	0.003	0.004
Black Tiger Prawn*	Aquaculture	116	110	6.4	0.014	0.022
Blue Sprat	Wild	269	95.8	17.2	0.003	0.004
Brown Lip Abalone	Wild	0.5	27.5	45.5	0.003	0.004
Brown Tiger Prawn	Wild	125	126	13.7	0.014	0.022
Burrowing Blackfish*	Wild	1.2	10	0.1	0.003	0.004
Endeavour Prawn	Wild	69.6	81.9	9.9	0.014	0.022
Green Lip Abalone	Wild	0.25	23.2	44.8	0.003	0.004
Green Lip Abalone	Aquaculture	12.5	40.3	41.7	0.003	0.004
Gummy Shark	Wild	112	19.3	9.1	0.071	0.110
Native Oyster	Aquaculture	201	143	17.3	0.009	0.015
Ocean Trout	Aquaculture	1,910	1,190	420	0.142	–
Pacific Oyster	Aquaculture	313	274	23	0.009	0.015
School Prawn	Wild	82.6	116	12.9	0.014	0.022
Southern Rock Lobster	Wild	51.8	93	10.1	0.014	0.022
Sydney Rock Oyster	Aquaculture	394	304	41.5	0.009	0.015
Western King Prawn	Wild	102	118	16.2	0.014	0.022
Yellowtail Kingfish	Aquaculture	873	994	275	0.071	–
Tuna, canned in brine†	Not reported	45	303	10	0.227	0.352
Tuna, yellowfin steaks grilled or bbq, brushed with olive oil†	Not reported	52	252	18	0.114	0.176

* Products were analysed cooked.

† Values taken from NUTTAB 2010.

### Theoretical dietary modelling: risk solver

The Risk Solver programme is an add-on feature to the Microsoft Excel computer programme (Risk Solver Premium V9.0.4.0 program; Frontline Systems, Inc.). The programme allows the random selection of foods from a list of pre-determined foods in which energy and nutrient profiles can be assessed. Diet simulation models were based on AGHE food group recommendations, and the food groups (iterations) that were included were usual food items consumed with percentage consumption frequencies based on the 1995 NNS (17). Seafood consumption frequencies were based on a recent Australian survey (18) that involved a similar age group to what the current theoretical models were performed against. Running the simulation procedure randomly selects each single food item according to the imputed number of daily serves in each food group (e.g. 5 random bread/cereal items from the list of 26, 2 random fruit items from the list of 22 fruits). This results in a mean intake of energy and nutrients for the whole day and for each food group. Based on a 14-day menu (varying in the number of serves for each food group/d and to reach overall AGHE recommendations), the simulation process runs the varying combinations 10,000 times (e.g. 10,000 combinations of 5 items of breads and cereals from the 26-item category) and mean energy and nutrient intakes are produced for each day and for the 14-day period. The likelihood (%) in which the theoretical diet would meet nutrient recommendations for a particular gender and/or age group can be calculated using estimated means calculated from the 14-day model.

### Model 1

The aim of Model 1 was to determine whether a simulation model which included a random selection of seafood types will meet the AI, SDT, and HF recommendation for LC *n*–3 PUFA in men and women 51–70 years of age, as well as the AMDR for protein, fat and carbohydrate, and AI for fibre. Consumption of seafood was entered at an average of 0.3 servings/d for women and 0.4 servings/d for men; over 1 week this reaches the recommendation of 2–3 servings (4). Each type of seafood was assigned a selection probability factor such that the more frequently consumed seafood (e.g. Atlantic salmon) was more likely to be selected than those less frequently consumed (e.g. abalone). Selection probability factors were estimated based on our recent survey (18) and are provided in Supplementary Tables 1–3. Due to the relatively frequent consumption of tuna (18), an option for canned tuna and for grilled/barbequed tuna was also included. The nutrient composition for tuna was taken from the NUTTAB 2010 database; therefore, a total of 26 seafood groups were available for selection in the modelling process. The number of servings for all other food categories was also entered over the 14 days according to AGHE recommendations (e.g. Day 1: 5 servings of breads and cereals, two servings of fruit; Day 2: 4 servings of breads and cereals, 2 servings of extras, etc.).

### Model 2

The aim of Model 2 was to determine whether including a random selection of the lower fat seafood (<10% total fat) will achieve LC *n*–3 PUFA recommendations for men and women aged 51–70 years in accordance with the AGHE. The seafood in Model 2 included Australian sardine, barramundi, blue sprat, burrowing blackfish, farmed prawns, wild prawns, gummy shark, oysters, southern rock lobster, and wild abalone, plus the two tuna options. A total of 23 lower fat seafood groups were included in the modelling process.

### Model 3 and Model 4

Two simulation models were undertaken to determine the number of servings of lower fat (Model 3) or higher fat seafood (Model 4) to meet LC *n*–3 PUFA recommendations from only seafood sources. Each lower or higher fat seafood was randomly included in the model in a continuous fashion until LC *n*–3 PUFA recommendations were met by 100% of men and women.

## Results

### Model 1: simulation of all seafood species

Appropriate numbers of food group servings from the AGHE were entered into a theoretical 14-day menu plan (Table 1), in which the numbers and types of foods from these food groups were randomly selected. The mean intake of energy, macronutrients and LC *n*–3 PUFA, and the percentage of men and women who would meet recommendations when including 0.3–0.4 servings/d (2–3 servings/week) of higher and lower fat seafood in an AGHE diet are described in Table 3. For women, including 2 servings/week of any type of seafood would result in 89% meeting the LC *n*–3 PUFA AI, while 66% of men would meet the AI with 3 servings/week. For both men and women, 37% would meet their respective SDT. The percentage of men and women that would meet the HF recommendation of 500 mg/d of combined EPA+DHA was 36%.

**Table 3:** Percentage of men and women aged 51–70 years who would meet energy, macronutrient, and long-chain omega 3 requirements from a theoretical 14-day AGHE plan (Model 1)

	Men		Women	
		
	Mean	%	Mean	%
Energy (kJ)	8,190	25^a^	7,448	59^b^
Protein (g)	93	100^c^	91	100^c^
CHO (g)	246	80^c^	213	67^c^
Sugar (g)	109	–	103	–
Fat (g)	58	77^c^	56	77^c^
Saturated fat (g)	24	40^c^	24	27^c^
Polyunsaturated fat (g)	10	–	9	–
Monounsaturated fat (g)	19	–	18	–
Fibre (g)	31	53^d^	25	52^d^
Total LC *n*–3 PUFA (mg)	585	66^d^; 37^e^	404	89^d^; 37^e^
EPA (mg)	267	–	182	–
DHA (mg)	235	–	160	–
DPA (mg)	83	–	62	–
EPA+DHA (mg)	502	36^f^	342	36^f^

AGHE: Australian Guide to Healthy Eating; LC *n*–3 PUFA: long-chain omega 3 polyunsaturated fatty acids; EPA: eicosapentanoic acid; DHA: docosahexanoic acid; DPA: docosapentanoic acid.

a Energy requirement for an average 51 to 70-year-old male, 1.7 m tall and with a physical activity level of 1.4

b Energy requirement for an average 51 to 70-year-old female, 1.6 m tall and with a physical activity level of 1.4

c AMDR: Acceptable Macronutrient Distribution

d AI: Adequate Intake: Women: 90 mg/d, Men 160 mg/d

e SDT: Suggested Dietary Target: Women 430 mg/d, Men 610 mg/d

f Heart Foundation recommendation in Australia (500 mg/d).

### Model 2: simulation of low-fat seafood into AGHE

Model 2 describes the mean intake of energy, macronutrients and LC *n*–3 PUFA following random selection of the lower fat seafood, at a frequency of 0.3 servings/d for women (2 servings/week) and 0.4 servings/d for men (3 servings/week) (Table 4). Eighty-three percent of women and 47% of men would achieve the AI for LC *n*–3 PUFA. Only 0.3 and 0.4% of men and women, respectively, would achieve the SDT and none would achieve the 500 mg/d HF recommendation.

**Table 4:** Percentage of men and women aged 51–70 years who would meet energy, macronutrient, and long-chain omega 3 requirements from a theoretical 14-day AGHE plan (Model 2).

	Men		Women	
		
	Mean	%	Mean	%
Energy (kJ)	8,080	19^a^	7,375	54^b^
Protein (g)	93	100^c^	91	100^c^
CHO (g)	246	83^c^	213	69^c^
Sugar (g)	109	–	103	–
Fat (g)	55	75^c^	54	77^c^
Saturated fat (g)	23	43^c^	24	28^c^
Polyunsaturated fat (g)	9	–	9	–
Monounsaturated fat (g)	18	–	17	–
Fibre (g)	31	53^d^	25	52^d^
Total LC *n*–3 PUFA (mg)	194	47^d^; 0.3^e^	143	83^d^; 0.4^e^
EPA (mg)	63	–	45	–
DHA (mg)	106	–	74	–
DPA (mg)	26	–	23	–
EPA+DHA (mg)	169	0.0%^f^	119	0.0%^f^

AGHE: Australian Guide to Healthy Eating; LC *n*–3 PUFA: long-chain omega 3 polyunsaturated fatty acids; EPA: eicosapentanoic acid; DHA: docosahexanoic acid; DPA: docosapentanoic acid.

a Energy requirement for an average 51 to 70-year-old male, 1.7 m tall and with a physical activity level of 1.4

b Energy requirement for an average 51 to 70-year-old female, 1.6 m tall and with a physical activity level of 1.4

c AMDR: Acceptable Macronutrient Distribution

d AI: Adequate Intake: Women: 90 mg/d, Men 160 mg/d

e SDT: Suggested Dietary Target: Women 430 mg/d, Men 610 mg/d

f Heart Foundation recommendation in Australia (500 mg/d).

### Model 3 and 4: simulation of low-fat seafood and high-fat seafood

Random selection of 100 g servings/week of lower fat seafood would provide approximately 54 mg/d of LC *n*–3 PUFA (Table 5). For women, 2.5 and 10 servings/week of lower fat seafood would be required for about 90% to meet the respective LC *n*–3 PUFA AI and SDT. For men, 4 and 14 servings/week of lower fat seafood would be required for about 90% to achieve the AI and SDT. In contrast, random selection of 100 g servings/week of higher fat seafood would provide 399 mg/d of LC *n*–3 PUFA, such that only 0.5 serving/week of higher fat seafood would be needed for men and women to meet the AI and only 1.5–2 servings/week to meet the SDT. In both men and women, consuming 2 servings/week of higher fat seafood would enable the HF recommendation of 500 mg/d to be met.

**Table 5:** Simulation to determine frequency of consumption of lower fat seafood and higher fat seafood to meet LC *n*–3 PUFA recommendations

	Lower fat seafood						Higher fat seafood					
		
Servings/week (100 g servings)	Mean LC *n*–3 PUFA (mg/d)	Percent achieving AI^a^ (%)		Percent achieving SDT^b^ (%)		Percent achieving 500 mg/d^c^ (%)	Mean LC *n*–3 PUFA (mg/d)	Percent achieving AI^a^ (%)		Percent achieving SDT^b^ (%)		Percent achieving 500 mg/d^c^ (%)
					
		Women	Men	Women	Men	Men and women		Women	Men	Women	Men	Men and women
1/2	27	0	0	0	0	0	200	100	100	0	0	0
1	54	14	0	0	0	0	399	100	100	56	0	0
1 1/2	81	35	2	0	0	0	599	100	100	100	26	70
2	109	61	8	0	0	0	799	100	100	100	100	100
2 1/2	136	94	22	0	0	0						
3	163	100	52	0	0	0						
3 1/2	191	100	71	0	0	0						
4	218	100	92	0	0	0						
4 1/2	246	100	99	0	0	0						
5	273	100	100	1	0	0						
5 1/2	300	100	100	2	0	0						
6	328	100	100	5	0	0						
7	382	100	100	22	0	1						
8	437	100	100	51	1	6						
9	491	100	100	80	5	23						
10	546	100	100	96	19	53						
11	600	100	100	100	43	81						
12	655	100	100	100	70	95						
13	710	100	100	100	89	99						
14	764	100	100	100	97	100						
15	818	100	100	100	100	100						

a AI: Adequate Intake: Women: 90 mg/d, Men 160 mg/d

b SDT: Suggested Dietary Target: Women 430 mg/d, Men 610 mg/d

c Heart Foundation recommendation in Australia (500 mg/d EPA+DHA).

## Discussion

The rationale behind the current dietary modelling was to investigate whether including the appropriate number of servings of seafood into national food consumption guidelines would achieve various recommendations for LC *n*–3 PUFA. We report that with a combination of higher and lower fat seafood at a frequency of 2 servings/week, the AI will be met by 89% of women and consumption of 3 servings/week will enable 66% of men to meet the AI. This type of modelling demonstrates that the consumption of a variety of seafood in a total diet based on national guidelines will result in at least two-thirds of older adults meeting the LC *n*–3 PUFA AI. However, when including only lower fat seafood into the diet, 83% of women will meet the AI but only 47% of men will meet it. Comparatively, 0.5 serving/week (50 g) of higher fat seafood is required for most men and women to meet the AI. These results suggest that meeting the AI for LC *n*–3 PUFA in women is possible, regardless of seafood choice, but it is likely to be a challenge for men, particularly when only lower fat seafood is preferred. Consumption of higher fat seafood appears a simple strategy to meet the AI for men and women on a regular basis.

Numerous agencies recommend 2–3 servings of oily fish per week to reduce risk for heart disease (4, 5, 9). The HF also sets out guidance for adult men and women to achieve the appropriate recommendations of omega 3 fatty acids by consuming various types of fish (4). The guidelines generally include species with higher amounts of LC *n*–3 PUFA such as fresh salmon, canned salmon and canned sardines in 150 g servings; however, it also includes varieties such as blue trevalla and gemfish (27). Our modelling demonstrates the potential to include the appropriate number of seafood into a usual weekly diet; however, the further recommendation of the *amount of fatty acids* to consume (i.e. a target of 500 mg/d EPA+DHA) produces a poorer result. This target would only be met by one-third of older adults when choosing a combination of higher and lower fat seafood, and the choice of only lower fat seafood would not enable any older adult to meet this target. In line with the guidelines, consumption of 2 servings of the higher fat seafood would enable 100% of men and women to meet the 500 mg/d recommendation. Therefore, for older adults wishing to reduce their risk of CHD, both a mixed seafood diet and a lower seafood diet does not appear to be an appropriate option; yet following the guidance of 2–3 servings of oily seafood per week is sufficient.

The NHMRC defines the SDT as a daily average intake from food and beverages for certain nutrients that may help in prevention of chronic disease (7). In our modelling of lower fat seafood, 14 servings/week and 10 servings/week would be required for men and women, respectively, to achieve the SDT. Comparatively, only 1.5–2 servings/week of higher fat seafood would be required to meet the SDT. This indicates that for individuals at risk of CVD, it would be extremely difficult to meet the SDT when the consumption of lower fat seafood only is desired. Additional strategies such as a fish oil supplement would be advised to meet the SDT.

It is clear from several studies in Australia (18, 20, 28) and overseas (13, 14, 29) that consumption of seafood is low. In addition, we (18), and others (17), have previously reported that lower fat seafood such as tuna and prawns are consumed in higher frequencies compared to higher fat seafood such as salmon and ocean trout. As a result of our modelling, consumption of lower fat seafood alone poses a problem for both men and women when trying to meet targets relevant to heart disease, and for men when trying to achieve the AI. Moreover, we demonstrated that for 90% of men to reach the AI from lower fat seafood alone, 4 servings/week would need to be consumed. For 90% of women to reach the AI, 2.5 servings/week of lower fat seafood would need to be consumed. This may not be feasible for a large proportion of the population on a weekly basis. Furthermore, when including a random selection of higher fat seafood, the percentage of adults who would meet these targets only slightly increased. In contrast, only 0.5 serving/week of higher fat seafood would be required for adults to meet the AI. Problematically, there are a limited variety of higher fat seafood species available on the Australian market (e.g. salmon, ocean trout, sardines, kingfish), thereby reducing choice in their selection. We also recently reported that in a sample of 854 older adults, only 38 and 18% indicated that salmon and trout were high in oils (fatty acids), respectively (19). Unpublished data from that survey also identified that 44% (*n*=378) believed they ate the right amount of finfish/seafood, 44% consumed finfish/seafood ≥2 times/week, but only 28% met the HF recommendation of 500 mg/d (4). Thus, there appears some disconnect regarding the types of seafood that need to be consumed in order to meet recommendations with what is actually being consumed.

Limitations to this analysis include that the modelling was ‘theoretical’, as food patterns were based on AGHE recommendations and were not based on typical dietary intakes for this age group. Therefore, the study results are not likely to be generalisable to all adults. However, the most recent nutrient intake data from Australia are the 1995 NNS (17). Results from that survey are outdated and food consumption patterns have changed (30). Dietary intake data from the most recent Australian NNS in 2010 were also not available; and the new Australian Dietary Guidelines were still in the draft form. Therefore, food consumption patterns were based on those most recently available. However, as a comparison to the draft guidelines, the number of servings of food groups from our modelling was not overly different (data not shown). Seafood types included in the study also were not representative of those available to consumers on the market, as compositional profiles were only available for a subset of all seafood, and did not include imported products. Therefore, other varieties of seafood that might also be consumed in Australia were not reflected in this data. However, tuna was included to limit this bias, and other seafood types that are frequently consumed such as barramundi and prawns were included in the analyses. Thus, we did capture a sound profile of seafood that could be included in a menu plan.

Based on the overall dietary modelling, it is evident that certain recommendations are difficult to achieve depending on the type of seafood chosen as well as the type of recommendation and reason for it to be achieved. This can clearly create confusion in the community when trying to determine individual targets for LC *n*–3 PUFA intake, or when individuals have a misunderstanding regarding the types of seafood to be consumed in order to meet targets. Altering the types of seafood from those higher in fat to only those lower in fat decreases the percentage of adults who would meet suggested targets. Our modelling provides evidence that consumption of 2–3 servings of higher fat seafood per week will meet any of the recommendations for LC *n*–3 PUFA intake, and therefore this recommendation appears to be most appropriate to communicate to the public. As previously discussed, many older adults do not recognise which types of seafood are higher in fatty acids, and therefore, in conjunction with the recommendation of 2–3 servings of fatty seafood, it is encouraged that guidance on the types of seafood to be consumed is highlighted. For those continuing to choose lower fat seafood, the option of a fish oil supplement would be required. Further messages and nutrition resources are needed which provide options on how to increase intake of LC *n*–3 PUFA, specifically through consumption of the higher fat seafood. This could be achieved through targeted education programmes, which could help inform a wide range of consumers about the consumption of seafood and meeting LC *n*–3 PUFA requirements.
